# Supplementary material for: Initiation and Single Dispensing in Cardiovascular and Insulin Medications: Prevalence and Explanatory Factors
Source: Int J Environ Res Public Health. 2020 May 12;17(10):3358. doi: 10.3390/ijerph17103358 (PMC7277594; doi:10.3390/ijerph17103358)
Supplement: Supplementary file 1 [file ijerph-17-03358-s001.zip › supplementary files/Supplementary Table S1.docx]

| Supplementary Table 1. Explanatory factors of non-initiation 3 months after prescription based on the bivariate analysis. | | | | | | | | | | | | | | | | | | | | | | | |
| --- | --- | --- | --- | --- | --- | --- | --- | --- | --- | --- | --- | --- | --- | --- | --- | --- | --- | --- | --- | --- | --- | --- | --- |
|  | | **Insulin**  **N=8,223** | | | | | **Antiplatelet**  **N=33,921** | | | | | | | **ACE Inhibitor**  **N=73,741** | | | | | **Statin**  **N=69,043** | | | | |
| **PATIENT VARIABLES** | | **Odds ratio (95% CI)** | | | **p-value** | | **Odds ratio (95% CI)** | | | | **p-value** | | | **Odds ratio (95% CI)** | | | **p-value** | | **Odds ratio (95% CI)** | | | | **p-value** |
| **Female patient (vs. male)** | | **0.843 (0.71; 0.99)** | | | **0.043** | | 0.965 (0.90; 1.04) | | | | 0.354 | | | 0.944 (0.89; 1.01) | | | 0.073 | | **0.882 (0.83; 0.94)** | | | | **0.001** |
| **Patient’s age** (continuous) | | **0.988 (0.98; 0.99)** | | | **0.001** | | **0.986 (0.98; 0.99)** | | | | **0.001** | | | **0.995 (0.99; 1.00)** | | | **0.001** | | **0.983 (0.98; 0.98)** | | | | **0.001** |
| **Patient’s SES^a^** | |  | | |  | |  | | | |  | | |  | | |  | |  | | | |  |
| **Urban 1 (lowest SES)** | | Ref. | | |  | | Ref. | | | |  | | | Ref. | | |  | | Ref. | | | |  |
| **Urban 2** | | 0.814 (0.61; 1.09) | | | 0.167 | | 0.994 (0.87; 1.14) | | | | 0.924 | | | **0.805 (0.72; 0.90)** | | | **0.001** | | **0.793 (0.72; 0.88)** | | | | **0.001** |
| **Urban 3** | | **0.719 (0.53; 0.97)** | | | **0.031** | | 0.954 (0.83; 1.10) | | | | 0.507 | | | **0.749 (0.67; 0.84)** | | | **0.001** | | **0.756 (0.68; 0.84)** | | | | **0.001** |
| **Urban 4** | | **0.726 (0.54; 0.98)** | | | **0.035** | | **0.845 (0.73; 0.98)** | | | | **0.022** | | | **0.671 (0.59; 0.76)** | | | **0.001** | | **0.685 (0.61; 0.76)** | | | | **0.001** |
| **Urban 5 (highest SES)** | | 0.807 (0.61; 1.08) | | | 0.146 | | 0.888 (0.76; 1.03) | | | | 0.121 | | | **0.693 (0.61; 0.79)** | | | **0.001** | | **0.683 (0.61; 0.76)** | | | | **0.001** |
| **Rural** | | 0.838 (0.63; 1.12) | | | 0.229 | | 1.071 (0.93; 1.23) | | | | 0.332 | | | **0.792 (0.70; 0.89)** | | | **0.001** | | **0.780 (0.70; 0.87)** | | | | **0.001** |
| **Patient’s place of origin** | |  | | |  | |  | | | |  | | |  | | |  | |  | | | |  |
| **Spain** | | Ref. | | |  | | Ref. | | | |  | | | Ref. | | |  | | Ref. | | | |  |
| **Americas** | | 1.380 (0.92; 2.08) | | | 0.120 | | **1.534 (1.27; 1.86)** | | | | **0.001** | | | **1.221 (1.05; 1.42)** | | | **0.008** | | **1.405 (1.23; 1.60)** | | | | **0.001** |
| **Asia/Oceania** | | **2.370 (1.40; 4.01)** | | | **0.001** | | **1.540 (1.07; 2.21)** | | | | **0.019** | | | **1.327 (1.03; 1.71)** | | | **0.027** | | **1.379 (1.07; 1.78)** | | | | **0.014** |
| **Europe outside Spain** | | 1.529 (0.84; 2.77) | | | 0.161 | | **2.105 (1.68; 2.63)** | | | | **0.001** | | | 1.188 (0.97; 1.45) | | | 0.091 | | **1.305 (1.09; 1.57)** | | | | **0.004** |
| **Africa** | | **1.605 (1.08; 2.39)** | | | **0.019** | | **1.353 (1.03; 1.77)** | | | | **0.027** | | | 1.175 (0.98; 1.41) | | | 0.081 | | 1.020 (0.83; 1.25) | | | | 0.850 |
| **BMI (cont.)** | | **0.957 (0.94; 0.97)** | | | **0.001** | | **0.972 (0.96; 0.98)** | | | | **0.001** | | | **0.976 (0.97; 0.98)** | | | **0.001** | | **0.982 (0.98; 0.99)** | | | | **0.001** |
| **≥1 New general prescriptions^a^ (vs. none)** | | **0.705 (0.56; 0.89)** | | | **0.004** | | **0.791 (0.71; 0.88)** | | | | **0.001** | | | **0.961 (0.89; 1.04)** | | | **0.330** | | **0.800 (0.74; 0.87)** | | | | **0.001** |
| **≥1 CV/diabetes new prescriptions^a^ (vs. none)** | | 0.810 (0.50; 1.31) | | 0.392 | | | | 0.921 (0.74; 1.15) | | 0.477 | | | 0.941 (0.75; 1.17) | | | 0.591 | | | | **0.786 (0.65; 0.95)** | | **0.014** | |
| **Number of visits^a^ (cont.)** | |  | |  | | | |  | |  | | |  | | |  | | | |  | |  | |
| **Visits to GP** | | **0.969 (0.95; 0.98)** | | **0.001** | | | | **0.962 (0.95; 0.97)** | | **0.001** | | | **0.994 (0.99; 1.00)** | | | **0.034** | | | | **0.965 (0.96; 0.97)** | | **0.001** | |
| **Visits to nurse** | | **0.975 (0.96; 0.99)** | | **0.001** | | | | **0.976 (0.97; 0.98)** | | **0.001** | | | 0.997 (0.99; 1.00) | | | 0.219 | | | | **0.974 (0.97; 0.98)** | | **0.001** | |
| **Active illnesses** | |  | |  | | | |  | |  | | |  | | |  | | | |  | |  | |
| **Pain** | | **0.717 (0.61; 0.85)** | | **0.001** | | | | **0.798 (0.74; 0.86)** | | **0.001** | | | **0.830 (0.78; 0.88)** | | | **0.001** | | | | **0.785 (0.74; 0.84)** | | **0.001** | |
| **Respiratory** | | 0.956 (0.75; 1.22) | | 0.717 | | | | **0.884 (0.79; 0.99)** | | **0.035** | | | 1.037 (0.94; 1.15) | | | 0.475 | | | | **0.833 (0.75; 0.93)** | | **0.001** | |
| **Physical disability^b^** | | 0.866 (0.73; 1.03) | | 0.105 | | | | **0.721 (0.66; 0.78)** | | **0.001** | | | 0.969 (0.90; 1.04) | | | 0.369 | | | | **0.751 (0.70; 0.81)** | | **0.001** | |
| **Cardiovascular conditions** | |  | |  | | | |  | |  | | |  | | |  | | | |  | |  | |
| Hypertension | | **0.579 (0.49; 0.68)** | | **0.001** | | | | **0.669 (0.62; 0.72)** | | **0.001** | | | **0.768 (0.72; 0.82)** | | | **0.001** | | | | **0.654 (0.61; 0.70)** | | **0.001** | |
|  | | **Insulin**  **N=8,223** | | | | | **Antiplatelet**  **N=33,921** | | | | | | | **ACE Inhibitor**  **N=73,741** | | | | | **Statin**  **N=69,043** | | | | |
| **PATIENT VARIABLES** | | **Odds ratio (95% CI)** | | | **p-value** | | **Odds ratio (95% CI)** | | | | **p-value** | | | **Odds ratio (95% CI)** | | | **p-value** | | **Odds ratio (95% CI)** | | | | **p-value** |
| Dyslipidemia | | **0.631 (0.53; 0.76)** | | **0.001** | | | | **0.792 (0.73; 0.86)** | | **0.001** | | | **0.851 (0.79; 0.91)** | | | **0.001** | | | | **0.915 (0.86; 0.97)** | | **0.004** | |
| Recent CVD (≤6 months) | | 1.221 (0.74; 2.02) | | 0.437 | | | | 0.849 (0.72; 1.00) | | 0.051 | | | **1.255 (1.04; 1.52)** | | | **0.018** | | | | **0.551 (0.44; 0.70)** | | **0.001** | |
| Established CVD (>6 months) | | 0.999 (0.83; 1.21) | | 0.993 | | | | **2.580 (2.38; 2.80)** | | **0.001** | | | **2.093 (1.94; 2.26)** | | | **0.001** | | | | **1.152 (1.05; 1.27)** | | **0.004** | |
| Alcohol and tobacco use^c^ | | 1.116 (0.93; 1.34) | | 0.235 | | | | 0.921 (0.84; 1.01) | | 0.070 | | | 0.937 (0.87; 1.01) | | | 0.085 | | | | 1.034 (0.97; 1.11) | | 0.319 | |
| **Mental disorders** | | **0.677 (0.55; 0.83)** | | **0.001** | | | | **0.785 (0.72; 0.86)** | | **0.001** | | | **0.924 (0.86; 0.99)** | | | **0.033** | | | | 0.937 (0.88; 1.00) | | 0.058 | |
| **Neurological** | | 0.917 (0.71; 1.18) | | 0.500 | | | | 0.964 (0.85; 1.09) | | 0.563 | | | 0.917 (0.82; 1.02) | | | 0.118 | | | | 0.903 (0.81; 1.00) | | 0.052 | |
| **Diabetes (1 and 2)** | | **0.336 (0.27; 0.42)** | | **0.001** | | | | **0.725 (0.66; 0.79)** | | **0.001** | | | **0.900 (0.83; 0.98)** | | | **0.010** | | | | **0.850 (0.79; 0.92)** | | **0.001** | |
| **Digestive system disorder** | | **0.708 (0.57; 0.89)** | | **0.003** | | | | **0.855 (0.78; 0.94)** | | **0.001** | | | 0.924 (0.85; 1.00) | | | 0.058 | | | | **0.809 (0.74; 0.88)** | | **0.001** | |
| **Urticaria/allergy** | | **1.857 (1.13; 3.06)** | | **0.015** | | | | 0.891 (0.69; 1.16) | | 0.388 | | | 0.986 (0.80; 1.22) | | | 0.896 | | | | 0.881 (0.72; 1.08) | | 0.231 | |
| **Hyper/hypothyroidism** | | 0.843 (0.62; 1.14) | | 0.271 | | | | **0.840 (0.73; 0.97)** | | **0.015** | | | 1.038 (0.93; 1.16) | | | 0.515 | | | | **0.864 (0.77; 0.96)** | | **0.009** | |
| Nº of comorbidities^d^ | | **0.808 (0.76; 0.86)** | | **0.001** | | | | **0.856 (0.83; 0.88)** | | **0.001** | | | **0.947 (0.93; 0.97)** | | | **0.001** | | | | **0.874 (0.86; 0.89)** | | **0.001** | |
|  | **Insulins (N=8,223)** | | | | | **Antiplatelets (N=33,921)** | | | | | | **ACE Inhibitors (N=73,741)** | | | | | | **Statins (N=69,043)** | | | | | |
| **PRESCRIBER VARIABLES** | **Odds ratio (95% CI)** | | **p-value** | | | **Odds ratio (95% CI)** | | | **p-value** | | | **Odds ratio (95% CI)** | | | **p-value** | | | **Odds ratio (95% CI)** | | | **p-value** | | |
| **Female GP (vs. male)** | 0.913 (0.77; 1.08) | | 0,295 | | | 0,959 (0,89; 1,04) | | | 0,293 | | | **0,900 (0,84; 0,96)** | | | **0,002** | | | 1,029 (0,97; 1,10) | | | 0,376 | | |
| **Age** (continuous) | 1.001 (0.99; 1.01) | | 0,865 | | | **1,008 (1,00; 1,01)** | | | **0,001** | | | **1,006 (1,00; 1,01)** | | | **0,001** | | | **1,005 (1,00; 1,01)** | | | **0,001** | | |
| **Substitute/resident GP (vs. other)** | 1.348 (0.92; 1.97) | | 0,122 | | | **0,791 (0,65; 0,97)** | | | **0,021** | | | **1,201 (1,04; 1,39)** | | | **0,013** | | | **1,202 (1,05; 1,37)** | | | **0,007** | | |
| **Teaching PC center (vs. regular)** | 0.949 (0.77; 1.17) | | 0,626 | | | 0,914 (0,81; 1,03) | | | 0,148 | | | 0,983 (0,88; 1,10) | | | 0,761 | | | 0,930 (0,85; 1,02) | | | 0,122 | | |

ACEI: ACE inhibitor; cont.: continuous variable; CVD: Cardiovascular disease; GP: General practitioner; SES: Socioeconomic status.

**Bold** numbers indicate statistically significant results.

^a^ In the 12 months preceding the index prescription.

^b^ Physical disabilities includes blindness, urinary incontinency and hypoacusia.

^c^ Alcohol or tobacco use was based on the qualitative appreciation of GPs; alcohol or tobacco use, though not cardiovascular conditions, are included as cardiovascular risk factors.

^d^ Number of comorbidities includes all the active illnesses listed in the table.
