# Supplementary material for: Initiation and Single Dispensing in Cardiovascular and Insulin Medications: Prevalence and Explanatory Factors
Source: Int J Environ Res Public Health. 2020 May 12;17(10):3358. doi: 10.3390/ijerph17103358 (PMC7277594; doi:10.3390/ijerph17103358)
Supplement: Supplementary file 1 [file ijerph-17-03358-s001.zip › supplementary files/Supplementary Table S2.docx]

Supplementary Table 2. Explanatory factors of non-initiation 6 months after prescription based on the bivariate analysis.

|  | Insulins (N= 8,223) | | Antiplatelets (N= 33,921) | | | ACE Inhibitors (N= 73,741) | | Statins (N= 69,043) | |
| --- | --- | --- | --- | --- | --- | --- | --- | --- | --- |
| PATIENT VARIABLES | **Odds ratio (95% CI)** | **p-value** | **Odds ratio (95% CI)** | **p-value** | | **Odds ratio (95% CI)** | **p-value** | **Odds ratio (95% CI)** | **p-value** |
| Female patient (vs. male) | **0.825 (0.69; 0.99)** | **0.041** | 0.955 (0.88; 1.03) | 0.254 | | 0.938 (0.88; 1.00) | 0.063 | **0.865 (0.81; 0.92)** | **0.001** |
| Patient’s age (continuous) | **0.986 (0.98; 0.99)** | **0.001** | **0.984 (0.98; 0.99)** | **0.001** | | **0.995 (0.99; 1.00)** | **0.001** | **0.982 (0.98; 0.98)** | **0.001** |
| Patient’s SES^a^ |  |  |  |  | |  |  |  |  |
| Urban 1 (lowest SES) | Ref. |  | Ref. |  | | Ref. |  | Ref. |  |
| Urban 2 | 0.706 (0.51; 0.97) | 0.034 | 0.992 (0.86; 1.14) | 0.910 | | **0.783 (0.70; 0.88)** | **0.001** | **0.784 (0.70; 0.87)** | **0.001** |
| Urban 3 | **0.637 (0.46; 0.88)** | **0.007** | 0.945 (0.82; 1.10) | 0.454 | | **0.711 (0.63; 0.80)** | **0.001** | **0.760 (0.68; 0.85)** | **0.001** |
| Urban 4 | **0.721 (0.53; 0.99)** | **0.042** | **0.849 (0.73; 0.99)** | **0.035** | | **0.633 (0.56; 0.72)** | **0.001** | **0.657 (0.59; 0.74)** | **0.001** |
| Urban 5 (highest SES) | 0.722 (0.53; 0.99) | 0.040 | 0.833 (0.71; 0.98) | 0.025 | | **0.675 (0.59; 0.77)** | **0.001** | **0.681 (0.60; 0.77)** | **0.001** |
| Rural | 0.754 (0.55; 1.03) | 0.073 | 1.062 (0.92; 1.23) | 0.421 | | **0.777 (0.69; 0.88)** | **0.001** | **0.782 (0.70; 0.87)** | **0.001** |
| Patient’s place of origin |  |  |  |  | |  |  |  |  |
| Spain | Ref. |  | Ref. |  | | Ref. |  | Ref. |  |
| Americas | 1.524 (0.99; 2.36) | 0.058 | **1.553 (1.27; 1.90)** | **0.001** | | **1.232 (1.05; 1.44)** | **0.009** | **1.280 (1.11; 1.48)** | **0.001** |
| Asia/Oceania | **2.321 (1.3; 4.14)** | **0.004** | **1.465 (0.99; 2.60** | **0.055** | | **1.242 (0.94; 1.63)** | **0.120** | **1.418 (1.08; 1.86)** | **0.011** |
| Europe outside Spain | 2.012 (1.11; 3.64) | 0.021 | **2.050 (1.61; 2.60)** | **0.001** | | 1.169 (0.94; 1.45) | 0.154 | **1.273 (1.05; 1.55)** | **0.016** |
| Africa | **1.702 (1.11; 2.62)** | **0.015** | **1.344 (1.01; 1.79)** | **0.044** | | 1.158 (0.95; 1.41) | 0.139 | 0.972 (0.78; 1.22) | 0.807 |
| BMI (cont.) | **0.959 (0.94; 0.98)** | **0.001** | **0.968 (0.96; 0.98)** | **0.001** | | **0.975 (0.97; 0.98)** | **0.001** | **0.979 (0.97; 0.99)** | **0.001** |
| ≥1 New prescriptions^a^ (vs. none) | **0.735 (0.57; 0.95)** | **0.020** | **0.793 (0.71; 0.89)** | **0.001** | | 0.955 (0.88; 1.04) | 0.296 | **0.774 (0.71; 0.85)** | **0.001** |
| ≥1 CV/diabetes new prescriptions^a^ (vs. none) | 0.765 (0.44; 1.33) | 0.343 | 0.953 (0.75; 1.21) | 0.691 | | 0.845 (0.66; 1.08) | 0.181 | **0.794 (0.65; 0.97)** | **0.028** |
| Number of visits^a^ (cont.) |  |  |  |  | |  |  |  |  |
| Visits to GP | **0.967 (0.95; 0.98)** | **0.001** | **0.957 (0.95; 0.96)** | **0.001** | | **0.994 (0.99; 1.00)** | **0.047** | **0.965 (0.96; 0.97)** | **0.001** |
| Visits to nurse | **0.976 (0.96; 0.99)** | **0.001** | **0.974 (0.97; 0.98)** | **0.001** | | 0.996 (0.99; 1.00) | 0.130 | **0.973 (0.97; 0.98)** | **0.001** |
| Active illnesses |  |  |  |  | |  |  |  |  |
| Pain | **0.671 (0.55; 0.81)** | **0.001** | **0.758 (0.70; 0.82)** | **0.001** | | **0.807 (0.75; 0.86)** | **0.001** | **0.771 (0.72; 0.82)** | **0.001** |
| Respiratory | 0.996 (0.76; 1.30) | 0.975 | **0.855 (0.76; 0.97)** | **0.013** | | 1.030 (0.93; 1.15) | 0.586 | **0.808 (0.72; 0.91)** | **0.001** |
| Physical disability^b^ | 0.866 (0.71; 1.05) | 0.145 | **0.703 (0.64; 0.77)** | **0.001** | | 0.961 (0.89; 1.04) | 0.299 | **0.735 (0.68; 0.80)** | **0.001** |
|  | **Insulins (N= 8,223)** | | **Antiplatelets (N= 33,921)** | | | **ACE Inhibitors (N= 73,741)** | | **Statins (N= 69,043)** | |
| PATIENT VARIABLES | **Odds ratio (95% CI)** | **p-value** | **Odds ratio (95% CI)** | **p-value** | | **Odds ratio (95% CI)** | **p-value** | **Odds ratio (95% CI)** | **p-value** |
| Cardiovascular conditions |  |  |  |  | |  |  |  |  |
| Hypertension | **0.596 (0.50; 0.72)** | **0.001** | **0.646 (0.60; 0.70)** | **0.001** | | **0.721 (0.67; 0.78)** | **0.001** | **0.636 (0.60; 0.68)** | **0.001** |
| Dyslipidemia | **0.595 (0.49; 0.73)** | **0.001** | **0.765 (0.70; 0.84)** | **0.001** | | **0.844 (0.78; 0.91)** | **0.001** | **0.919 (0.86; 0.98)** | **0.011** |
| Recent CVD (≤6 months) | 0.814 (0.42; 1.57) | 0.538 | 0.793 (0.66; 0.95) | 0.012 | | **1.26 (1.03; 1.54)** | **0.024** | **0.575 (0.45; 0.74)** | **0.001** |
| Established CVD (>6 months) | 0.949 (0.77; 1.17) | 0.629 | **2.534 (2.32; 2.76)** | **0.001** | | **2.084 (1.92; 2.26)** | **0.001** | **1.186 (1.07; 1.31)** | **0.001** |
| Alcohol and tobacco use^c^ | 1.124 (0.92; 1.37) | 0.253 | 0.903 (0.82; 0.99) | 0.036 | | 0.940 (0.87; 1.02) | 0.123 | 1.021 (0.95; 1.10) | 0.565 |
| Mental disorders | **0.628 (0.50; 0.79)** | **0.001** | **0.777 (0.71; 0.85)** | **0.001** | | **0.928 (0.86; 1.00)** | **0.060** | 0.917 (0.85; 0.99) | 0.019 |
| Neurological | 0.924 (0.70; 1.22) | 0.580 | 0.896 (0.78; 1.03) | 0.115 | | 0.905 (0.80; 1.02) | 0.094 | 0.864 (0.77; 0.97) | 0.011 |
| Diabetes (1 and 2) | **0.320 (0.25; 0.41)** | **0.001** | **0.688 (0.62; 0.76)** | **0.001** | | **0.874 (0.80; 0.95)** | **0.002** | **0.816 (0.75; 0.89)** | **0.001** |
| Digestive system disorder | **0.678 (0.53; 0.87)** | **0.003** | **0.832 (0.75; 0.92)** | **0.001** | | 0.901 (0.83; 0.98) | 0.020 | **0.771 (0.70; 0.84)** | **0.001** |
| Urticaria/allergy | **1.948 (1.14; 3.33)** | **0.015** | 0.852 (0.64; 1.13) | 0.273 | | 0.928 (0.73; 1.17) | 0.531 | 0.930 (0.75; 1.16) | 0.514 |
| Hyper/hypothyroidism | 0.986 (0.72; 1.36) | 0.930 | **0.844 (0.73; 0.98)** | **0.026** | | 1.009 (0.89; 1.14) | 0.883 | **0.877 (0.78; 0.99)** | **0.027** |
| Nº of comorbidities^d^ | **0.799 (0.75; 0.85)** | **0.001** | **0.834 (0.81; 0.86)** | **0.001** | | **0.934 (0.91; 0.96)** | **0.001** | **0.861 (0.84; 0.88)** | **0.001** |
|  | **Insulins (N= 8,223)** | | **Antiplatelets (N= 33,921)** | | | **ACE Inhibitors (N= 73,741)** | | **Statins (N= 69,043)** | |
| PRESCRIBER VARIABLES | **Odds ratio (95% CI)** | **p-value** | **Odds ratio (95% CI)** | | **p-value** | **Odds ratio (95% CI)** | **p-value** | **Odds ratio (95% CI)** | **p-value** |
| Female GP (vs. male) | 0.929 (0.77; 1.12) | 0.443 | 0.944 (0.87; 1.03) | | 0.176 | **0.865 (0.81; 0.93)** | **0.001** | 1.005 (0.94; 1.08) | 0.874 |
| Age (continuous) | 1.001 (0.99; 1.01) | 0.829 | **1.008 (1.00; 1.01)** | | **0.001** | **1.006 (1.00; 1.01)** | **0.001** | **1.006 (1.00; 1.01)** | **0.001** |
| Substitute/resident GP (vs. other) | 1.395 (0.92; 2.11) | 0.113 | **0.792 (0.64; 0.98)** | | **0.033** | **1.205 (1.03; 1.41)** | **0.017** | **1.276 (1.11; 1.47)** | **0.001** |
| Teaching PC center (vs. regular) | 1.033 (0.83; 1.29) | 0.773 | 0.901 (0.79; 1.02) | | 0.108 | 0.972 (0.87; 1.09) | 0.628 | 0.926 (0.84; 1.02) | 0.122 |

ACEI: ACE inhibitor; cont.: continuous variable; CVD: Cardiovascular disease; GP: General practitioner; SES: Socioeconomic status.

**Bold** numbers indicate statistically significant results.

^a^ In the 12 months preceding the index prescription.

^b^ Physical disabilities includes blindness, urinary incontinency and hypoacusia.

^c^ Alcohol or tobacco use was based on the qualitative appreciation of GPs; alcohol or tobacco use, though not cardiovascular conditions, are included as cardiovascular risk factors.

^d^ Number of comorbidities includes all the active illnesses listed in the table.
