# Supplementary material for: Initiation and Single Dispensing in Cardiovascular and Insulin Medications: Prevalence and Explanatory Factors
Source: Int J Environ Res Public Health. 2020 May 12;17(10):3358. doi: 10.3390/ijerph17103358 (PMC7277594; doi:10.3390/ijerph17103358)
Supplement: Supplementary file 1 [file ijerph-17-03358-s001.zip › supplementary files/Supplementary Table S3.docx]

Supplementary Table 3. Explanatory factors of single dispensing within a 3-months period based on the bivariate analysis.

|  | **Insulins (N= 8,223)** | | **Antiplatelets (N= 33,921)** | | **ACE Inhibitors (N= 73,741)** | | **Statins (N= 69,043)** | |
| --- | --- | --- | --- | --- | --- | --- | --- | --- |
| **PATIENT VARIABLES** | **Odds ratio (95% CI)** | **p-value** | **Odds ratio (95% CI)** | **p-value** | **Odds ratio (95% CI)** | **p-value** | **Odds ratio (95% CI)** | **p-value** |
| **Female patient (vs. male)** | 1.075 (0.95; 1.22) | 0.249 | **1.225 (1.15; 1.30)** | **0.001** | **1.141 (1.10; 1.18)** | **0.001** | 0.986 (0.94; 1.04) | 0.568 |
| **Patient’s age** (continuous) | 0.997 (0.99; 1.00) | 0.171 | **0.973 (0.97; 0.98)** | **0.001** | **0.988 (0.99; 0.99)** | **0.001** | **0.978 (0.98; 0.98)** | **0.001** |
| **Patient’s SES^a^** |  |  |  |  |  |  |  |  |
| Urban 1 (lowest SES | Ref. |  | Ref. |  | Ref. |  | Ref. |  |
| Urban 2 | 1.128 (0.90; 1.42) | 0.299 | 0.947 (0.85; 1.06) | 0.330 | 1.000 (0.93; 1.07) | 0.989 | 1.010 (0.93; 1.10) | 0.821 |
| Urban 3 | 0.879 (0.70; 1.11) | 0.280 | 0.939 (0.84; 1.05) | 0.267 | 1.006 (0.94; 1.08) | 0.859 | 0.967 (0.88; 1.06) | 0.451 |
| Urban 4 | 0.963 (0.77; 1.21) | 0.743 | 0.917 (0.82; 1.03) | 0.132 | 0.976 (0.91; 1.05) | 0.511 | 0.941 (0.86; 1.03) | 0.185 |
| Urban 5 (highest SES) | 1.152 (0.92; 1.44) | 0.208 | 0.934 (0.83; 1.05) | 0.252 | 1.031 (0.96; 1.11) | 0.422 | 0.925 (0.84; 1.02) | 0.106 |
| Rural | 1.043 (0.84; 1.30) | 0.708 | 1.008 (0.90; 1.12) | 0.882 | 0.952 (0.88; 1.03) | 0.195 | 1.002 (0.92; 1.09) | 0.957 |
| **Patient’s place of origin** |  |  |  |  |  |  |  |  |
| Spain | Ref. |  | Ref. |  | Ref. |  | Ref. |  |
| Americas | 1.329 (0.97; 1.83) | 0.081 | **1.744 (1.48; 2.05)** | **0.001** | **1.375 (1.26; 1.50)** | **0.001** | **1.736 (1.56; 1.93)** | **0.001** |
| Asia/Oceania | **1.772 (1.10; 2.85)** | **0.019** | **2.291 (1.71; 3.06)** | **0.001** | **1.631 (1.40; 1.89)** | **0.001** | **1.523 (1.24; 1.88)** | **0.001** |
| Europe outside Spain | 1.454 (0.90; 2.34) | 0.123 | **1.776 (1.43; 2.21)** | **0.001** | **1.678 (1.50; 1.88)** | **0.001** | **1.792 (1.56; 2.06)** | **0.001** |
| Africa | **1.936 (1.44; 2.61)** | **0.001** | **1.901 (1.54; 2.35)** | **0.001** | **1.277 (1.15; 1.42)** | **0.001** | **1.437 (1.24; 1.67)** | **0.001** |
| **BMI (cont.)** | **0.976 (0.96; 0.99)** | **0.001** | **0.989 (0.98; 0.99)** | **0.001** | **0.992 (0.99; 1.00)** | **0.001** | **0.984 (0.98; 0.99)** | **0.001** |
| **≥1 New prescriptions^a^ (vs. none)** | **1.205 (1.03; 1.40)** | **0.017** | **1.235 (1.14; 1.33)** | **0.001** | **1.098 (1.05; 1.15)** | **0.001** | **1.104 (1.04; 1.17)** | **0.001** |
| **≥1 CV/diabetes new prescriptions^a^ (vs. none)** | 1.128 (0.82; 1.56) | 0.464 | 0.933 (0.77; 1.12) | 0.468 | **0.832 (0.73; 0.95)** | **0.008** | 0.868 (0.75; 1.01) | 0.063 |
| **Number of visits^a^ (cont.)** |  |  |  |  |  |  |  |  |
| **Visits to GP** | 1.005 (1.00; 1.01) | 0.267 | **0.990 (0.98; 1.00)** | **0.001** | 0.997 (0.99; 1.00) | 0.132 | **0.978 (0.97; 0.98)** | **0.001** |
| **Visits to nurse** | 0.999 (0.99; 1.01) | 0.850 | **0.993 (0.99; 1.00)** | **0.002** | **0.990 (0.99; 0.99)** | **0.001** | **0.972 (0.97; 0.98)** | **0.001** |
| **Active illnesses** |  |  |  |  |  |  |  |  |
| **Pain** | **0.842 (0.74; 0.95)** | **0.007** | 0.944 (0.89; 1.01) | 0.073 | 0.964 (0.93; 1.00) | 0.058 | **0.928 (0.88; 0.97)** | **0.003** |
| **Respiratory** | 1.140 (0.96; 1.36) | 0.137 | **0.814 (0.74; 0.90)** | **0.001** | 0.964 (0.91; 1.02) | 0.234 | **0.835 (0.76; 0.91)** | **0.001** |
| **Physical disability^b^** | 0.990 (0.87; 1.13) | 0.884 | **0.738 (0.69; 0.79)** | **0.001** | **0.897 (0.86; 0.94)** | **0.001** | **0.756 (0.71; 0.80** | **0.001** |
|  |  |  |  |  |  |  |  |  |
|  |  |  |  |  |  |  |  |  |
|  | **Insulins (N= 8,223)** | | **Antiplatelets (N= 33,921)** | | **ACE Inhibitors (N= 73,741)** | | **Statins (N= 69,043)** | |
| **PRESCRIBER VARIABLES** | **Odds ratio (95% CI)** | **p-value** | **Odds ratio (95% CI)** | **p-value** | **Odds ratio (95% CI)** | **p-value** | **Odds ratio (95% CI)** | **p-value** |
| **Cardiovascular conditions** |  |  |  |  |  |  |  |  |
| Hypertension | **0.775 (0.68; 0.88)** | **0.001** | **0.564 (0.53; 0.60)** | **0.001** | **0.701 (0.67; 0.73)** | **0.001** | **0.622 (0.59; 0.65)** | **0.001** |
| Dyslipidemia | **0.779 (0.68; 0.89)** | **0.001** | **0.756 (0.71; 0.81)** | **0.001** | **0.813 (0.78; 0.85)** | **0.001** | **0.912 (0.87; 0.96)** | **0.001** |
| Recent CVD (≤6 months) | 1.217 (0.83; 1.79) | 0.320 | **0.526 (0.46; 0.60)** | **0.001** | **0.778 (0.69; 0.88)** | **0.001** | **0.618 (0.52; 0.74)** | **0.001** |
| Established CVD (>6 months) | 1.005 (0.87; 1.16) | 0.942 | **0.533 (0.48; 0.59)** | **0.001** | **0.860 (0.81; 0.91)** | **0.001** | **0.776 (0.71; 0.85)** | **0.001** |
| Alcohol and tobacco use^c^ | 0.934 (0.81; 1.07) | 0.341 | 0.987 (0.92; 1.06) | 0.727 | 0.958 (0.92; 1.00) | 0.054 | 1.031 (0.98; 1.09) | 0.268 |
| **Mental disorders** | 0.883 (0.77; 1.02) | 0.086 | **0.909 (0.85; 0.98)** | **0.008** | 0.974 (0.93; 1.02) | 0.225 | 0.967 (0.92; 1.02) | 0.233 |
| **Neurological** | 0.904 (0.75; 1.09) | 0.298 | 1.055 (0.95; 1.17) | 0.303 | 0.993 (0.93; 1.06) | 0.829 | 0.937 (0.86; 1.02) | 0.120 |
| **Diabetes (1 and 2)** | **0.387 (0.31; 0.48)** | **0.001** | **0.564 (0.52; 0.61)** | **0.001** | **0.665 (0.63; 0.70)** | **0.001** | **0.689 (0.65; 0.74)** | **0.001** |
| **Digestive system disorder** | 1.107 (0.95; 1.29) | 0.184 | **0.888 (0.82; 0.96)** | **0.002** | 0.998 (0.95; 1.05) | 0.920 | **0.876 (0.82; 0.94)** | **0.001** |
| **Urticaria/allergy** | 1.000 (0.62; 1.62) | 1.000 | 1.022 (0.83; 1.26) | 0.837 | **1.193 (1.06; 1.34)** | **0.004** | 1.109 (0.95; 1.29) | 0.185 |
| **Hyper/hypothyroidism** | 0.962 (0.77; 1.19) | 0.725 | 0.984 (0.88; 1.10) | 0.771 | 0.938 (0.88; 1.01) | 0.075 | 0.972 (0.89; 1.06) | 0.512 |
| Nº of comorbidities^d^ | **0.927 (0.89; 0.97)** | **0.001** | **0.845 (0.83; 0.86)** | **0.001** | **0.928 (0.92; 0.94)** | **0.001** | **0.889 (0.87; 0.91)** | **0.001** |
|  | **Insulins (N= 8,223)** | | **Antiplatelets (N= 33,921)** | | **ACE Inhibitors (N= 73,741)** | | **Statins (N= 69,043)** | |
| **PRESCRIBER VARIABLES** | **Odds ratio (95% CI)** | **p-value** | **Odds ratio (95% CI)** | **p-value** | **Odds ratio (95% CI)** | **p-value** | **Odds ratio (95% CI)** | **p-value** |
| **Female GP (vs. male)** | 1.133 (0.99; 1.29) | 0.061 | 0.987 (0.92; 1.05) | 0.694 | 0.992 (0.95; 1.03) | 0.700 | **0.877 (0.83; 0.92)** | **0.001** |
| **Age** (continuous) | 0.996 (0.99; 1.00) | 0.218 | 1.000 (1.00; 1.00) | 0.984 | 1.001 (1.00; 1.00) | 0.250 | **1.005 (1.00; 1.01)** | **0.001** |
| **Substitute/resident GP (vs. other)** | 1.222 (0.90; 1.66) | 0.195 | **1.314 (1.14; 1.51)** | **0.001** | **1.373 (1.26; 1.50)** | **0.001** | **1.276 (1.15; 1.42)** | **0.001** |
| **Teaching PC center (vs. regular)** | 0.954 (0.83; 1.10) | 0.525 | **0.919 (0.85; 1.00)** | **0.048** | 0.950 (0.89; 1.01) | 0.089 | **0.889 (0.83; 0.95)** | **0.001** |

ACEI: ACE inhibitor; cont.: continuous variable; CVD: Cardiovascular disease; GP: General practitioner; SES: Socioeconomic status.

**Bold** numbers indicate statistically significant results.

^a^ In the 12 months preceding the index prescription.

^b^ Physical disabilities includes blindness, urinary incontinency and hypoacusia.

^c^ Alcohol or tobacco use was based on the qualitative appreciation of GPs; alcohol or tobacco use, though not cardiovascular conditions, are included as cardiovascular risk factors.

^d^ Number of comorbidities includes all the active illnesses listed in the table.
