# Supplementary material for: Initiation and Single Dispensing in Cardiovascular and Insulin Medications: Prevalence and Explanatory Factors
Source: Int J Environ Res Public Health. 2020 May 12;17(10):3358. doi: 10.3390/ijerph17103358 (PMC7277594; doi:10.3390/ijerph17103358)
Supplement: Supplementary file 1 [file ijerph-17-03358-s001.zip › supplementary files/Supplementary Table S4.docx]

Supplementary Table 4. Explanatory factors of non-initiation within a 3-months period based on the multivariate analysis.

|  | **Insulins^a^ (N= 8,223)** | | **Antiplatelets^b^ (N= 33,921)** | | **ACE Inhibitors^c^ (N= 73,741)** | | **Statins^d^ (N= 69,043)** | |
| --- | --- | --- | --- | --- | --- | --- | --- | --- |
| **PATIENT VARIABLES** | **Odds ratio (95% CI)** | **p-value** | **Odds ratio (95% CI)** | **p-value** | **Odds ratio (95% CI)** | **p-value** | **Odds ratio (95% CI)** | **p-value** |
| **Constant** | 0.390 (0.20;0.76) | 0.006 | 0.571 (0.41;0.79) | 0.001 | 0.149 (0.12;0.19) | 0.001 | 0.312 (0.24;0.41) | 0.001 |
| **Female patient (vs. male)** | 0.992 (0.82;1.20) | 0.933 | ─ | ─ | ─ | ─ | 0.982 (0.92;1.05) | 0.593 |
| **Patient’s age (cont.)** | 0.996 (0.99;1.00) | 0.277 | **0.986 (0.98;0.99)** | **0.001** | ─ | ─ | **0.987 (0.98;0.99)** | **0.001** |
| **Patient’s SES^a^** |  |  |  |  |  |  |  |  |
| Urban 1 (lowest SES) | Ref. |  | Ref. |  | Ref. |  | Ref. |  |
| Urban 2 | 0.727 (0.52;1.01) | 0.055 | 1.029 (0.89;1.19) | 0.696 | **0.790 (0.70;0.89)** | **0.001** | **0.801 (0.72;0.89)** | **0.001** |
| Urban 3 | **0.654 (0.47;0.91)** | **0.012** | 0.978 (0.84;1.14) | 0.773 | **0.725 (0.64;0.82)** | **0.001** | **0.775 (0.69;0.87)** | **0.001** |
| Urban 4 | 0.753 (0.55;1.04) | 0.085 | 0.916 (0.79;1.07) | 0.268 | **0.647 (0.57;0.74)** | **0.001** | **0.679 (0.60;0.76)** | **0.001** |
| Urban 5 (highest SES) | 0.730 (0.53;1.01) | 0.055 | 0.884 (0.75;1.04) | 0.140 | **0.685 (0.60;0.78)** | **0.001** | **0.701 (0.62;0.79)** | **0.001** |
| Rural | 0.791 (0.58;1.08) | 0.145 | 1.149 (0.99;1.33) | 0.066 | **0.768 (0.68;0.87)** | **0.001** | **0.800 (0.72;0.89)** | **0.001** |
| **Patient’s place of origin** |  |  |  |  |  |  |  |  |
| Spain | Ref. |  | Ref. |  | Ref. |  | Ref. |  |
| Americas | 1.254 (0.80;1.96) | 0.321 | **1.324 (1.07;1.63)** | **0.008** | **1.270 (1.08;1.49)** | **0.003** | 1.108 (0.96;1.28) | 0.171 |
| Asia/Oceania | **1.824 (1.00;3.32)** | **0.049** | 1.090 (0.73;1.63) | 0.672 | 1.267 (0.96;1.67) | 0.093 | 1.202 (0.92;1.58) | 0.184 |
| Europe outside Spain | 1.611 (0.88;2.94) | 0.121 | **1.535 (1.20;1.96)** | **0.001** | 1.166 (0.94;1.45) | 0.164 | 1.092 (0.90;1.33) | 0.384 |
| Africa | 1.324 (0.85;2.07) | 0.218 | 1.111 (0.83;1.49) | 0.486 | 1.213 (1.00;1.48) | 0.054 | 0.861 (0.69;1.08) | 0.193 |
| **BMI (cont.)** | **0.974 (0.96;0.99)** | **0.003** | **0.976 (0.97;0.98)** | **0.001** | **0.982 (0.97;0.99)** | **0.001** | **0.990 (0.98;1.00)** | **0.003** |
| **≥1 New prescriptions^a^ (vs. none)** | 0.796 (0.61;1.04) | 0.090 | 0.937 (0.84;1.05) | 0.264 | ─ | ─ | **0.819 (0.74;0.90)** | **0.001** |
| **≥1 CV/diabetes new prescriptions^a^ (vs. none)** | ─ | ─ | ─ | ─ | ─ | ─ | 1.072 (0.86;1.34) | 0.543 |
| **Number of visits^a^ (cont.)** |  |  |  |  |  |  |  |  |
| **Visits to GP** | 0.994 (0.98;1.01) | 0.492 | **0.974 (0.97;0.98)** | **0.001** | ─ | ─ | **0.984 (0.98;0.99)** | **0.001** |
| **Visits to nurse** | 0.989 (0.97;1.00) | 0.117 | 0.995 (0.99;1.00) | 0.164 | ─ | ─ | 0.998 (0.99;1.00) | 0.544 |
| **Active illnesses** |  |  |  |  |  |  |  |  |
| **Pain** | 0.860 (0.70;1.06) | 0.148 | 0.965 (0.89;1.05) | 0.418 | **0.827 (0.77;0.89)** | **0.001** | **0.916 (0.85;0.98)** | **0.013** |
| **Respiratory** | ─ | ─ | 0.937 (0.82;1.06) | 0.319 | ─ | ─ | 0.937 (0.83;1.06) | 0.297 |
|  | **Insulins^a^ (N= 8,223)** | | **Antiplatelets^b^ (N= 33,921)** | | **ACE Inhibitors^c^ (N= 73,741)** | | **Statins^d^ (N= 69,043)** | |
| **PATIENT VARIABLES** | **Odds ratio (95% CI)** | **p-value** | **Odds ratio (95% CI)** | **p-value** | **Odds ratio (95% CI)** | **p-value** | **Odds ratio (95% CI)** | **p-value** |
| **Physical disability**^b^ | ─ | ─ | **0.878 (0.80;0.97)** | **0.009** | ─ | ─ | **0.913 (0.84;0.99)** | **0.038** |
| **Cardiovascular conditions** |  |  |  |  |  |  |  |  |
| Hypertension | 0.807 (0.65;1.00) | 0.051 | **0.811 (0.74;0.89)** | **0.001** | **0.752 (0.70;0.81)** | **0.001** | **0.750 (0.70;0.81)** | **0.001** |
| Dyslipidemia | **0.685 (0.56;0.84)** | **0.001** | **0.838 (0.76;0.92)** | **0.001** | **0.859 (0.79;0.93)** | **0.001** | ─ | ─ |
| Recent CVD (≤6 months) | ─ | ─ | 0.851 (0.71;1.02) | 0.083 | **1.261 (1.03;1.54)** | **0.024** | **0.652 (0.51;0.84)** | **0.001** |
| Established CVD (>6 months) | ─ | ─ | **3.146 (2.87;3.45)** | **0.001** | **2.262 (2.08;2.46)** | **0.001** | **1.572 (1.41;1.75)** | **0.001** |
| **Mental disorders** | **0.756 (0.59;0.96)** | **0.024** | **0.840 (0.76;0.93)** | **0.001** | ─ | ─ | ─ | ─ |
| **Neurological** | ─ | ─ | ─ | ─ | ─ | ─ | 0.923 (0.82;1.03) | 0.169 |
| **Diabetes (1 and 2)** | ─ | ─ | **0.786 (0.71;0.87)** | **0.001** | **0.85 (0.78;0.93)** | **0.001** | 0.965 (0.88;1.05) | 0.421 |
| **Digestive system disorder** | 0.798 (0.62;1.04) | 0.091 | 0.973 (0.88;1.08) | 0.615 | ─ | ─ | **0.880 (0.80;0.97)** | **0.007** |
| **Urticaria/allergy** | **2.236 (1.30;3.85)** | **0.004** | ─ | ─ | ─ | ─ | ─ | ─ |
| **Hyper/hypothyroidism** | ─ | ─ | 0.957 (0.82;1.11) | 0.570 | ─ | ─ | 0.951 (0.84;1.07) | 0.408 |
|  | **Insulins^a^ (N= 8,223)** | | **Antiplatelets^b^ (N= 33,921)** | | **ACE Inhibitors^c^ (N= 73,741)** | | **Statins^d^ (N= 69,043)** | |
| **PRESCRIBER VARIABLES** | **Odds ratio (95% CI)** | **p-value** | **Odds ratio (95% CI)** | **p-value** | **Odds ratio (95% CI)** | **p-value** | **Odds ratio (95% CI)** | **p-value** |
| **Female GP (vs. male)** | ─ | ─ | ─ | ─ | **0.870 (0.81;0.93)** | **0.001** | ─ | ─ |
| **Substitute/resident GP (vs. other)** | ─ | ─ | **0.804 (0.65;1.00)** | **0.049** | **1.260 (1.08;1.47)** | **0.003** | **1.281 (1.11;1.47)** | **0.001** |

ACEI: ACE inhibitor; cont.: continuous variable; CVD: Cardiovascular disease; GP: General practitioner; SES: Socioeconomic status.

**Bold** numbers indicate statistically significant results.

‘─’ indicates that the factor was not selected to be included in the multivariate analysis based on results from the bivariate analysis.

^a^ In the 12 months preceding the index prescription.

^b^ Physical disabilities includes blindness, urinary incontinency and hypoacusia.
